# The Interplay of Exosomes and NK Cells in Cancer Biology

**DOI:** 10.3390/cancers13030473

**Published:** 2021-01-26

**Authors:** Inês A. Batista, Sofia T. Quintas, Sónia A. Melo

**Affiliations:** 1 i3S—Instituto de Investigação e Inovação em Saúde, Universidade do Porto, 4200-135 Porto, Portugal; ibatista@ipatimup.pt (I.A.B.); squintas@ipatimup.pt (S.T.Q.); 2 IPATIMUP—Institute of Molecular Pathology and Immunology of University of Porto, 4200-135 Porto, Portugal; 3 ICBAS—Instituto de Ciências Biomédicas de Abel Salazar, University of Porto, 4050-313 Porto, Portugal; 4 FMUP—Faculty of Medicine, University of Porto, 4200-319 Porto, Portugal

**Keywords:** cancer, exosomes, tumor microenvironment, Natural Killer cells, immune system, immunomodulation

## Abstract

**Simple Summary:**

Cancer is a lethal disease and the second most common cause of death worldwide. It is estimated that the next decades will bring a rise of about 50% in cancer incidence and mortality. At present, immunotherapy has shown the best results for cancer treatment. Because of this, the study of the anti-tumor immune response has gained a significant importance in cancer research. Many are the factors described to be involved in this process. Exosomes are extracellular vesicles that shape the cellular program of the recipient cells and are involved in cancer progression. Our review explores the link between exosomes and natural killer (NK) cells, the possible consequences in cancer progression, and the enclosed potential to identify new immunotherapy targets within this crosstalk.

**Abstract:**

Natural killer (NK) cells are innate lymphoid cells involved in tumor surveillance. These immune cells have the potential to fight cancer growth and metastasis, as such, their deregulation can result in tumor immune escape. Recently exosomes were described as mediators of intercellular communication between cancer and NK cells. The exact role of this subclass of extracellular vesicles (EVs), which transport genetic and molecular material to recipient cells, in NK cell biology in the context of cancer, is still an open question. Several reports have demonstrated that tumor-derived exosomes (TDEs) can exert immunomodulatory activities, including immunosuppression, thus promoting cancer progression. Some reports demonstrate that the interplay between cancer exosomes and NK cells allows tumors to escape immune regulation. On the other hand, tumor exosomes were also described to activate NK cells. Additionally, studies show that NK cell exosomes can modulate the immune system, opening up their potential as an immunotherapeutic strategy for cancer treatment. Our review will focus on the reprogramming effect of cancer exosomes on NK cells, and the immunotherapeutic potential of NK cells-derived exosomes.

## 1. Introduction

In 2020, nearly 19 million people will be diagnosed with cancer and an estimated number of 10 million cancer deaths will occur around the globe [1,2]. Alarmingly, these numbers are estimated to rise to 29.5 million and 16.4 million, respectively, by 2040 [2]. Hence, the demand for new and more effective treatments has never been more critical. Immunotherapy has shown the best results in cancer treatment [3,4]. This therapeutic strategy aims to enable the recognition of tumor cells by the patient’s immune system and, thus, promote their elimination. However, many known and unknown factors limit immunotherapy applicability to all cancer types, as well as to the percentage of patients that respond favorably [5,6]. A thorough understanding of the role of immune cells in tumor progression is the necessary path to overcome these challenges.

The tumor microenvironment (TME) comprehends cancer cells together with endothelial cells, fibroblasts, immune cells, perivascular cells, adipocytes, and also the extracellular matrix [7,8]. The survival and proliferation of tumor cells, as well as their migratory and invasion capacity, vastly depend on their ability to communicate with and reprogram their microenvironment [8]. Exosomes have emerged as mediators of the intercellular communication between tumor cells and the TME. Extensive evidence shows that cancer cells strategically use exosomes to boost tumor progression and metastasis, promote angiogenesis, mediate changes in the tumor landscape that allow the tumor to escape the immune system, and resist therapy [9,10,11,12,13,14].

Tumor cells decrease antigen presentation to evade immune surveillance [15,16,17]. This is accomplished through the downregulation of the pathways involved in antigen processing and presentation machinery, including the downregulation of expression of the major histocompatibility complex I (MHC-I) [18,19]. The ability of T cells to recognize tumor cells is then compromised and natural killer (NK) cells enter into play [20,21]. In brief, NK cells are able to recognize these MHC-I-lacking tumor cells, becoming activated upon interaction with stress-induced ligands found on tumor cells, which triggers NK cell-mediated tumor cell killing [22]. In this review, we discuss the modulation of the activity of NK cells by cancer exosomes and the implications that this knowledge can have on potential new immunotherapeutic strategies. We address the main limitations faced in the field, including the paradoxical roles of cancer exosomes on the regulation of NK cells’ function. In addition, we highlight the need for further studies that can clarify where NK cells exert their effects, at the tumor site or at the periphery, as well as to the fact that the majority of the studies are still limited to the use of cell culture supernatants and need to be validated in vivo and/or in human samples.

## 2. NK Cells in Cancer Killing

### 2.1. The Immune Response to Cancer Cells

When the organism senses an infection caused by a foreign pathogen, the immune system is activated. The same holds true in the presence of cancer cells [23,24]. A robust anti-tumor immune response is mounted in order to neutralize the threat. Yet, tumors manage to escape this anti-tumor immune response, and are able to survive and proliferate [23,25].

Cancer immunoediting is a concept involving three phases, which explain how the immune system can both be responsible for the prevention of tumor development (elimination phase) and for editing the tumor immunogenicity and ultimately promote tumor progression (equilibrium and escape phases) [24,25,26]. The three phases associated with cancer immunoediting are:

1. Elimination (or cancer immune surveillance): The immune system recognizes and eliminates transformed cells before a clinically relevant tumor develops. NK cells were shown to be particularly relevant in this phase in preclinical models, which were more prone to develop tumors when NK cells were depleted or dysfunctional [27,28,29]. Furthermore, individuals with a decreased natural cytotoxic activity and impaired NK cells’ activity presented a higher risk of developing cancer [30,31,32,33] and of postoperative recurrence [34]. When the immune system fails to completely eliminate tumor cells, these enter into a temporary, yet dynamic equilibrium state [25,26,35].
2. Equilibrium: During this phase the immune system constrains tumor growth, and an equilibrium is established between tumor elimination by the immune system and tumor growth [25];
3. Escape: The immune system is no longer able to eliminate tumor cells, which in turn grow into a clinically apparent tumor. This ability to escape the immune system is owed to the selection of tumor variants, during the equilibrium phase, that are less immunogenic and express immunosuppressive pathways [25]. Unfortunately, cancer diagnosis occurs after cancer cells have gone through all the steps of the cancer immunoediting process [36], and when tumor-infiltrating immune cells, including NK cells, already present an altered phenotype [37,38,39]. Interestingly, these changes in NK cells’ phenotype can include the downregulation of its activating receptors due to overstimulation by their ligands, which are strategically overexpressed by tumor cells. Examples include natural killer group 2 member D ligands (NKG2DLs), the DNAX Accessory Molecule-1 (DNAM1) ligands CD155 and CD112, as well as ligands of natural cytotoxicity receptors (NCRs), such as NKp30, NKp44, and NKp46, as reviewed in [40].

### 2.2. Natural Killer Cells

The great majority of the existing anti-cancer immunotherapeutic approaches are focused on T cell activation and T cell-mediated elimination of cancer cells [41,42,43]. In recent years, the potential of NK cells in cancer killing has attracted the attention of the scientific community. In fact, NK cells were found to be able to exert cytotoxic effects against several malignancies [44,45]. NK cell-based immunotherapies have shown promising clinical results, with minimal safety concerns [46,47]. Therefore, the study of NK cells’ function and regulation in cancer, namely via their interaction with cancer exosomes, is crucial for the future development of effective immunotherapeutic strategies.

#### 2.2.1. Origin of NK Cells

**Figure 1:**
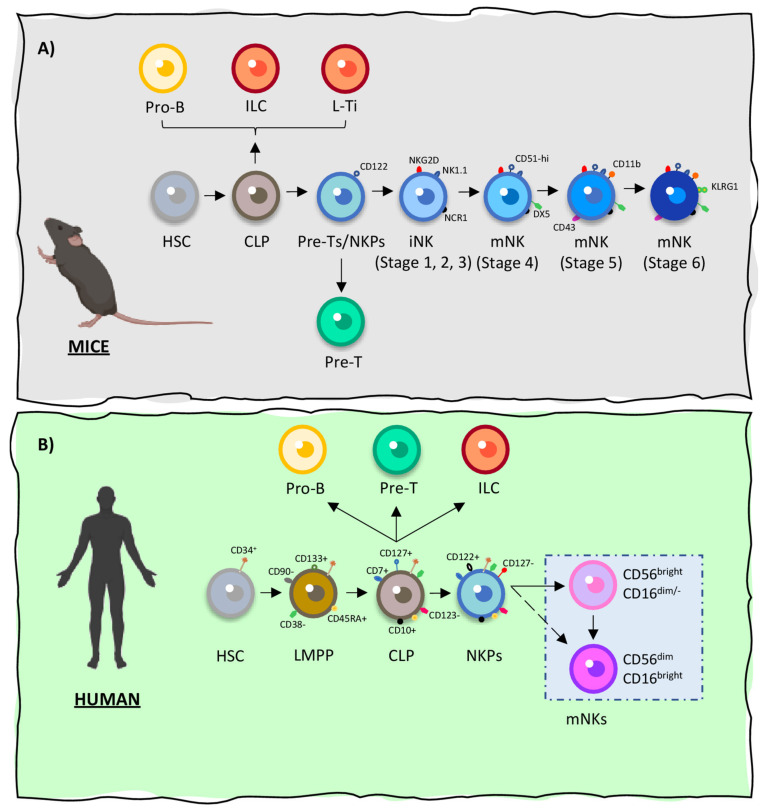
Natural killer (NK) cells’ origin in mice and humans. In both species, natural killer (NK) cells have their cellular origin in oligopotent common lymphoid progenitor cells (CLPs). (**A**) In mice, NK precursors (NKP) evolve into mature NK (mNK) cells through a 6-stage process, in which they acquire the expression of specific activating and inactivating receptors. CLP cells develop from hematopoietic stem cells (HSCs). CLPs then give rise to Pro-B cells, innate lymphoid cells (ILCs), Pre-T cells (L-Ti) and CD122+ Pre-T/early NKP lineages within the murine bone marrow. The transition of NKPs into committed immature NK cells (iNK) is marked by the expression of NKG2D (stage 1), followed by the expression of NK1.1 and NCR1 (stages 2 and 3). iNKs differentiate into mNKs, acquiring the expression of CD51, DX5, CD43 and CD11b (stages 4 and 5). Finally, mNKs migrate into secondary lymphoid organs (stage 6). (**B**) In humans, CLPs also suffer differentiation to originate mNKs through a complex process in which differential and sequential regulation of key surface receptors takes place. CD122 (IL2Rβ) sets the fate of CLPs into the NK lineage. The resultant NKPs then differentiate into mNKs, which can be divided into different subpopulations. These subpopulations of mNK cells are defined by the acquisition and differential expression of CD16 and/or CD56. This process ultimately gives origin to CD56dim/-CD16bright NK cells and to CD56bright CD16dim NK cells, the most common NK cells in humans (around 90% and 10%, respectively).

The different steps of NK cells maturation take place in the bone marrow of both humans and mice, being defined by the differential expression of lineage-specific surface markers by NK cell progenitors as illustrated on Figure 1 [44]. In addition, human NK cells have also been shown to mature in secondary lymphoid organs [44,48]. After becoming fully mature, NK cells present the capacity of self-renewal and are thought to persist in the host’s immune system for months or even years [49]. A detailed representation of the process of NK cells development in both species is illustrated on Figure 1A (mice) and Figure 1B (human) according to the information found on the following references [44,45,48,50,51].

#### 2.2.2. Natural Killer Cells: Function

**Figure 2:**
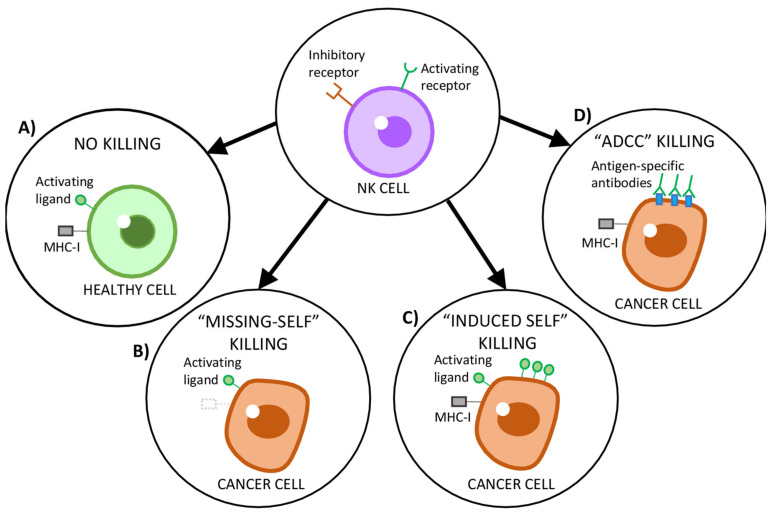
Schematic representation of natural killer (NK) cells’ recognition strategies of target cells. (**A**) NK cells’ activity is regulated by a variety of signaling molecules that engage the activating and inhibitory receptors on the surface of NK cells. Healthy cells express the major histocompatibility complex class I (MHC-I), which suppresses NK cells activation, as well as low levels of activating ligands. (**B**) Tumor cells downregulate MHC-I in order to escape cytotoxic T cells. However, this prompts NK cells to recognize and kill such tumor cells through their “missing-self” program. (**C**) Tumor cells upregulate stress ligands, which are recognized by activating receptors on NK cells, countermanding any inhibitory signals and inducing NK cell-mediated cytotoxicity. (**D**) Antibody-dependent cell-mediated cytotoxicity (ADCC) is triggered by CD16 (one of the most potent activating receptors on NK cells), which recognizes the antibodies bound to antigen-coated tumor cells.

NK cells, contrarily to T and B cells, do not require prior encounter with an antigen to rapidly recognize and kill target cells and, thus, are considered part of the innate immune system. The functional activity of NK cells is regulated by germ-line-coded activating and inhibitory receptors present on their plasma membrane (Figure 2), which are able to distinguish “non-self” cells (including neoplastic cells) from “self” cells based on the detection of specific molecules on the cells’ surface [44,45,52,53]. A key molecule for NK cells regulation and tolerance towards “self” cells is the antigen-presenting protein MHC-I, which is found on the surface of most nucleated “self” cells of the body [20,21]. Upon recognition of MHC-I by inhibitory receptors (KIR in humans and Ly49 in mice, which work in concert with CD94/NKG2A) present on the membrane of NK cells, an inhibitory signal is generated to block the activation of NK cells, which remain in a “Non-killing” state (Figure 2A) [20,21,45]. On the other hand, the downregulation of MHC-I, which is often observed in tumor cells, triggers NK cells’ activation. This mechanism is known as “Missing-self” recognition (Figure 2B) [20,21,45,49,52,54]. In addition, “non-self” cells often overexpress activating ligands on their surface, such as NKG2DLs or CD70 (the ligand for CD27) [55]. Furthermore, tumor cells also release some of these ligands into their microenvironment. For example, platelet-derived growth factor DD (PDGF-DD) has been shown to be released by many types of tumors and to activate the NK cells’ receptor NKp44, stimulating the secretion of interferon-γ (IFN-γ), tumor necrosis factor-α (TNF-α) and chemokines to trigger tumor cell–cycle arrest [56]. This overexpression of specific ligands of NK cells’ activating receptors countermands any inhibitory signals, triggering the “Induced-self killing” program on NK cells and the subsequent lysis of target cells (Figure 2C) [44,45]. Another program of cell killing mediated by NK cells is the antibody-dependent cell-mediated cytotoxicity (ADCC) mediated by CD16, the only receptor on NK cells that is capable of activating resting NK cells when stimulated alone [57]. The ADCC process is initiated when CD16 recognizes the constant (Fc) region of immunoglobulins present on the surface of target cells (Figure 2), allowing NK cells to identify and kill antibody-coated cancer cells (Figure 2D) [57]. Remarkably, polymorphisms of CD16 have been shown to enhance its affinity for anti-CD20 monoclonal antibody (Rituximab) and, thus, improve the clinical response in patients with B-cell lymphomas [58,59,60].

The described mechanisms allow NK cells to recognize tumor cells and mediate tumor cell killing either through: (1) The release of cytotoxic granules containing granzyme and perforin, which cause cancer cell lysis [44,45]; or, (2) the secretion of cytokines (e.g., IFN-γ and TNF-α) and chemokines (e.g., chemokine C-C motif ligand 3 (CCL3), CCL4 and CCL5) into the extracellular microenvironment, which trigger tumor cell apoptosis and can shape the immune response of other cells of the immune system [55,61].

#### 2.2.3. NK Cells: A Part of the Innate Immune System, of the Adaptive Immune System or Both?

As mentioned, NK cells are commonly considered part of the first line of defense against pathogens—the innate immune system [62]. However, in the past decade, increasing evidence shows that NK cells can acquire a memory-like phenotype upon activation [45,63,64,65,66,67], being able to carry out a robust secondary immune response [63,65,66,68].

## 3. Exosomes

Exosomes are a subclass of extracellular vesicles (EVs) of endocytic origin, [9,10,69] characterized by their small size (from 30 to 150 nm) [10,13,70,71]. During exosomes’ biogenesis, several molecules, such as DNA, mRNAs, long noncoding RNAs (lncRNA), microRNAs (miRNA), lipids, and proteins are packed into the lumen of exosomes [10]. According to the Exocarta, researchers have identified 9769 proteins, 3408 mRNAs, and 2838 miRNAs in exosomes [72]. The packing of these molecules is influenced by many factors, including the cell of origin and its pathophysiological state [9]. It comes as no surprise that the resultant heterogeneous populations of exosomes released by different cells act as platforms for the regulation of complex physiological and pathological processes [73], including cancer [10,69]. Exosomes are also known for their ability to travel long distances [74,75], having been identified in a wide variety of body fluids, including blood [76], ascites [77], saliva [78], urine [79], semen [80], cerebrospinal fluid [81], and breast milk [82]. These characteristics denote the importance of exosomes as vehicles of biological information, being able to reprogram neighbor or distant recipient cells [74,75], and explains their involvement across the various hallmarks of cancer [9,10,11,12,13,14].

### 3.1. Exosomes Biogenesis, Secretion, and Uptake

**Figure 3:**
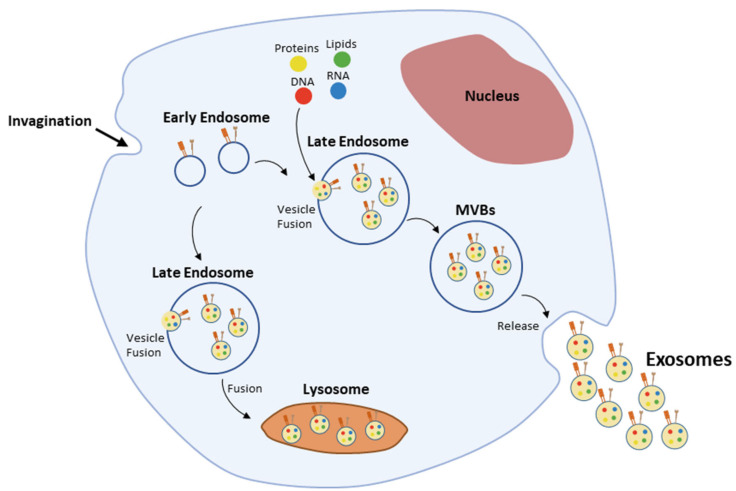
Exosomes biogenesis.

Exosomes biogenesis (Figure 3) is a multistep process [10], initiated upon inward membrane invagination, followed by the formation of early endosomes [11,83]. Inside the cell, early endosomes mature into late endosomes—also called multivesicular bodies (MVBs)—upon the inward budding of clathrin-coated domains in their membrane and consequent accumulation of intraluminal vesicles (ILVs) in their lumen [83,84]. The formation of ILVs is accompanied by the differential sorting of proteins, lipids and nucleic acids present in the cell’s cytosol or in the endosomes’ membrane [84,85]. These processes are driven by the endosomal sorting complexes required for transport (ESCRT; reviewed in Henne et al. [85]). Nevertheless, endosomes maturation into MVBs and cargo sorting still occur within mammalian cells in the absence of the ESCRT machinery, implying the existence of other ESCRT-independent pathways [86], which have been shown to involve lipids, tetraspanins, and heat shock proteins (Hsp) often found in exosomes [87,88,89,90,91]. Rab GTPases, such as Rab11 and Rab35, have also been shown to be involved in this early stage of exosomes biogenesis [12,92]. Later on, fusion of MVBs with the plasma membrane and secretion of exosomes into the extracellular compartment is controlled by Rab27a and Rab27b [93].

After their release from the cell of origin, exosomes can travel towards recipient cells, being able to transmit complex messages and change the behavior of those cells via three main mechanisms: (1) Interaction with receptors on the cell’s surface, which trigger the modulation of intracellular signaling pathways; (2) fusion with the plasma membrane and release of their cargo into the cell’s cytosol; and (3) endocytosis by phagocytosis, macropinocytosis or receptor-mediated endocytosis. The mechanism used by cells to uptake exosomes seems to be independent of the origin of the exosomes. However, further studies are needed in order to clarify whether exosomes from a specific cell of origin are preferably taken up through a specific mechanism and how this can influence their function [94].

### 3.2. Exosomes Modulate the Tumor Immune Landscape

Exosomes participate in a dynamic and bidirectional flow of information between cancer cells and cells of the TME. This plays a decisive role in tumor progression and metastasis formation in great part by modulating the anti-tumor immune response through local action within the TME and/or at distant lymphoid organs [83,95].

The first evidence of exosomes’ potential effects on tumor progression showed that exosomes derived from immune cells might enhance the activity of other immune cells against tumor cells and, thus, have a tumor suppressive role. Back in 1996 Raposo et al. [96] discovered that MHC-II is present at the surface of exosomes derived from human Epstein-Barr Virus-transformed B cells. The well-known link between MHC-I and MHC-II, and the presentation of foreign antigens to CD8^+^ and CD4^+^ T cells, respectively, resulting in their activation and maturation, sparked the attention over the possible biological functions of exosomes [97,98]. After the observation in 1998 that exosomes derived from dendritic cells (DCs) pre-exposed to cancer peptides are able to promote a cytotoxic immune response against tumor cells in vivo [99], the interest on this topic began to grow [75]. Since then, various other reports show that DCs release exosomes that can activate B, T and NK cells [99,100,101,102,103,104,105,106]. On the other hand, exosomes derived from immune cells were shown to promote tumor progression. Cai et al. demonstrated that activated CD8^+^ T cells-derived exosomes were able to promote the migratory and invasive capacities of melanoma and lung cancer cells through the combined activation of ERK and nuclear factor-kappa B (NFκB) signaling pathways [107].

The interaction between tumor-derived exosomes (TDEs) and the immune system has also been described in the literature. Some of the initial evidences on the interaction between TDEs and the immune system indicate that TDEs are immunostimulatory, delivering tumor antigens to DCs that then present them to cytotoxic T cells and activate a robust immune response [108,109,110]. Lancaster and Febbraio conducted a study in which they show that TDEs present the stress protein Hsp70 on their surface [111]. Moreover, subsequent studies have shown that Hsp70-expressing exosomes promote the activation of NK cell-mediated cancer killing [111,112,113,114,115], as further described on Section 4.1. Several other studies implicate TDEs on the activation [40,116,117,118] as well as on the inhibition of NK cells [119,120,121,122,123,124,125,126,127,128]. This will be further reviewed in the Section 4.

Overwhelming evidence show an immunosuppressive role for TDEs, suggesting a dual function in the modulation of anti-tumor immune response. Yu et al. showed that treatment of mammary tumor-bearing mice with TDEs lead to the accumulation of myeloid precursors cells in the spleen and inhibition of their differentiation into DCs by promoting the transcription of interleukin-6 (IL-6) [129]. In prostate cancer, TDEs expressing Fas ligand (FasL) decreased T cell proliferation and induced T cell apoptosis in a dose-dependent manner [130]. Another study revealed that TDEs carrying transforming growth factor beta 1 (TGFβ1) altered IL-2 responsiveness, leading to an increased action of regulatory T cells (Tregs) in detriment of cytotoxic cells [131]. Nasopharyngeal carcinoma-derived exosomes were shown to be enriched in miRNAs that target and inhibit the MAPK1 and JAK/STAT pathways in T cells, impairing their function and proliferation and inducing the differentiation of Tregs, being associated with the upregulation of exosomal miRNAs, which down-regulate the MAPK1 and JAK/STAT pathways [132]. TDEs were also shown to promote a switch in the differentiation of myeloid cells into myeloid-derived suppressor cells (MDSCs), thus accelerating the process of lung metastasis in a MyD88-dependent manner [133,134]. Furthermore, it was observed in pancreatic cancer that exosomal miRNAs may also inhibit the immune response by downregulation of MHC-II transcription in DCs [135]. Also in pancreatic cancer, TDEs express miR-203, which downregulate the expression of toll-like receptor 4 (TLR4), IL-12, and TNF-α in DCs [136]. This contributes to the inhibition of DC-mediated antigen presentation to cytotoxic immune cells following the treatment with miR-203-positive TDEs [136].

Put together, these studies suggest that TDEs play an immunomodulatory role in cancer, being able to target both the effector and antigen-presenting arms of the immune system. Even though a small number of studies show that TDEs can stimulate the immune system [97,98], they are predominantly considered immunosuppressive. Further efforts should focus on uncovering the immunomodulatory mechanisms that are set in motion by TDEs and on how these can be targeted to boost the patient’s own immune system to eliminate cancer.

## 4. Exosomes and NK Cells: How Does This Interplay Influence Cancer Progression?

In recent years, increasing evidence supporting TDEs’ pivotal role in the regulation of the TME has been provided, denoting the modulation of the tumor immune landscape by TDEs to favor tumor progression [137,138]. NK cells were shown to be able to interact with and uptake PKH67-labelled exosomes derived from human tumor cells, such as pancreatic cancer (L3.6pl) [123], myeloid leukemia (K562), T cell leukemia (Jurkat), hepatoblastoma (HepG2), cervical cancer (HeLa), breast carcinoma (MCF-7) [139], and multiple myeloma (SKO-007-subclone J3) [140] cells. Additionally, Huyan et al. found that the capacity of NK cells to uptake exosomes was dependent on the tumor cell of origin [139]. These results highlight the potential for TDEs to function as immunomodulators, being key players in the transmission of information between tumor cells and NK cells to regulate NK cells’ function for tumor benefit. In contrast with DCs-derived exosomes [101,102,103,104,105], TDEs are mostly described as having immunosuppressive roles in cancer [83,131,141,142,143]. Nevertheless, some reports indicate that TDEs may also take part in the activation of cytotoxic immune cells, either by presenting antigens to DCs, that then trigger CD8^+^ T cells activation [110], or by directly activating NK cells [112,113].

### 4.1. Tumor Exosomes as Mediators of NK Cells’ Activation

**Figure 4:**
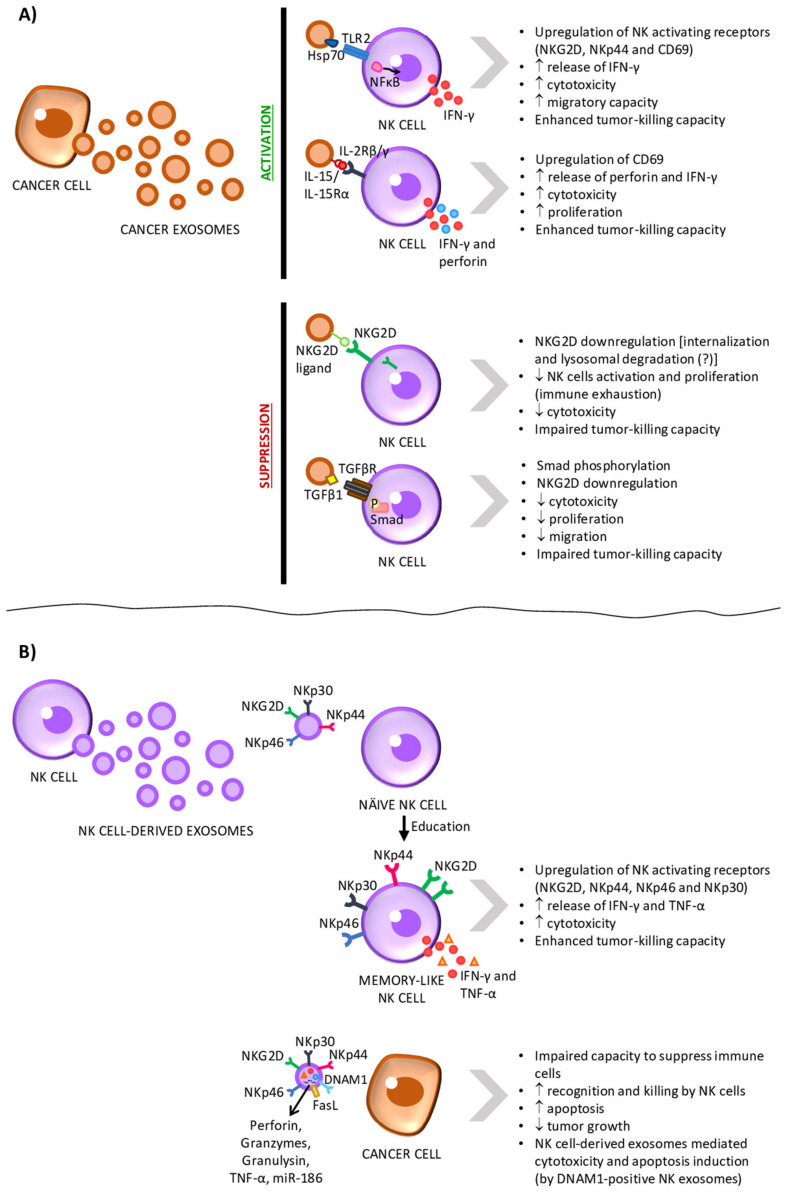
Exosomes mediate the interplay of information between tumor cells and natural killer (NK) cells. (**A**) Exosomes derived from tumor cells have been shown to express different signaling proteins on their surface (heat-shock protein 70 (Hsp70) and the interleukin-15(IL-15)/IL-15 receptor α (IL-15Rα) complex), thus promoting the activation of NK cells’ function. Nevertheless, studies show that the prolonged exposure to tumor-derived exosomes led to NK cells exhaustion. This suppressive effect seems to be driven by exosomal natural killer group 2 member D (NKG2D) ligands and transforming growth factor beta 1 (TGFβ1), which downregulate NKG2D expression and reduce the cytotoxicity, proliferation and migratory capacity of NK cells. (**B**) Exosomes derived from NK cells have been shown to overexpress the NK activating receptors NKp30, NKp44, NKp46, and NKG2D and to be able to educate naïve NK cells, which evolve into a memory-like state, with increased cytotoxicity and enhanced tumor-killing capacity. NK-derived exosomes also express the ligand of the apoptosis receptor Fas (FasL) and carry perforin, granzymes A and B, granulysin, TNF-α, and miR-186, which regulate the tumor’s capacity to modulate the immune system for its benefit and induce tumor lysis. DNAX Accessory Molecule-1 (DNAM1) is also found on the surface of NK cell-derived exosomes, promoting tumor cells death.

Exosomes derived from tumor cells have been shown to have the capacity to induce NK cells’ activity (Figure 4A and Table 1). This immune-stimulatory role of TDEs seems to be dependent on Hsp proteins, as discussed in this section. Gastpar et al. [112] have shown that Hsp70/Bcl-2–associated athanogene 4 (Bag4)-positive exosomes derived from human pancreatic (Colo357) and colon (CX2) cancer cell lines were able to stimulate the migratory capacity and cytolytic activity of NK cells isolated from human peripheral blood mononuclear cells. These effects were reversed by Hsp70 antibody treatment. Similar results were observed when NK cells isolated from either SCID mice or 129Sv immunocompetent mice were treated with Hsp70-positive exosomes from the melanoma cell line Ge [113]. In this study, Elsner et al. also found that NK cells’ cytotoxic activity induced by Hsp70-positive exosomes was dependent on the expression of NKG2DLs on the surface of target tumor cells. In vitro experiments showed that Hsp70-positive exosomes from Hsp-70 overexpressing melanoma cells were capable of activating mice’s NK cells. Activated NK cells could than eliminate YAC-1 cells, which expressed NKG2DLs in a constitutively manner, and NKG2DL-overexpressing human melanoma cells [113]. Interestingly, it has been shown that treatment of human hepatocarcinoma HepG2 cells with chemotherapeutic drugs to which they are resistant (i.e., carboplatin or irinotecan hydrochloride) increases exosomes production and the expression of Hsp70, Hsp90, and Hsp60 on exosomes’ surface. These exosomes were able to stimulate the cytotoxicity of NK cells derived from human peripheral blood mononuclear cells (PBMCs) as measured by the enhanced expression of CD69, NKG2D, and NKp44 receptors and downregulation of CD94 on the surface of NK cells, as well as by the release of granzyme B [114]. In line with these findings, J558-derived exosomes overexpressing Hsp70 boosted CD8^+^ T cells’ and NK cells’ cytotoxicity in BALB/c mice and protected all mice injected subcutaneously with J558 mouse myeloma cells from tumor development. In fact, treatment with anti-CD8 or anti-NK neutralizing antibodies, such as anti-NK1.1 (clone PK136), significantly abrogated the protection offered by Hsp70-overexpressing exosomes [115]. Nevertheless, Hsp70-positive myeloma exosomes derived from SKO-007-subclone J3 cells did not affect human PBMCs-derived NK cells’ cytotoxicity or the release of cytolytic granules. Instead, these exosomes activated the TLR2/ NFκB signaling pathway through Hsp70 and induced the production of the cytokine IFN-γ [140].

Apart from Hsp proteins, IL-15 and IL-15Rα were also shown to be expressed on TDEs isolated from multiple myeloma cells, promoting human NK cells proliferation. However, the effects of IL-15/IL-15Rα-expressing TDEs on NK cells’ cytotoxic activity was not studied [144].

In summary, these findings strongly suggest that Hsp-expressing exosomes boost the cytotoxic immune response against cancer by playing a role in NK cells’ recruitment and activation. As Hsp proteins are stress-induced proteins involved in protein refolding and stabilization, they can thus promote tumor progression and resistance to chemotherapy through the stabilization of oncogenic proteins [116]. In the context of immunosurveillance, exosomal Hsp proteins have been shown to have both immune stimulatory and immune suppressive effects, as reviewed in [117]. Thus, the evident importance of these proteins for exosomal-mediated immune modulation, as well as their potential as diagnostic markers and as therapeutic targets in cancer, call for further investigation of the specific molecular interactions and pathways involved in such processes. Furthermore, an in vivo confirmatory study of Hsp-expressing exosomes’ stimulatory role in NK recruitment and activation should be conducted in the future. On the other hand, it is important to understand if the observed NK cells’ activation is maintained after long term exposure of NK cells to TDEs. As discussed in the next section, it has been shown that prolonged stimulation of activating receptor NKG2D on the surface of NK cells leads to its downregulation and inhibition of NK cells activity [40]. Additionally, Li et al. [118] showed that treatment of human PBMCs-derived NK cells with exosomes from K562 myeloid leukemia cells engineered to overexpress IL-15, IL-18, and 4-1BBL enhances NK cells’ killing activity, the release of perforin and the production of IFN-γ. However, after long exposure (i.e., more than 48 h) to TDEs, a decrease in NK cells’ cytotoxicity, a higher expression of the inhibitory receptor NKG2A, and a lower expression of the activating receptors NKG2D and NKp44 were detected [118]. This suggests that the immune activation mediated by TDEs might, later on, trigger immune cell exhaustion as a result of prolonged exposure to stimulatory signals.

### 4.2. Tumor Exosomes as Mediators of NK Cell Suppression

NK cells’ activity is regulated by the integration of a variety of signals through their activating and inhibitory receptors [44,53]. As mentioned above, NK cells are tolerant towards the host cells due to the ubiquitous expression of MHC-I, which interacts with inhibitory receptors on the surface of NK cells [20,21]. However, in order to evade cytotoxic T cells that recognize mutated antigens presented by MHC-I on the cells’ surface, tumor cells downregulate MHC-I expression [18]. This, in turn, can lead to the recognition and killing of tumor cells by NK cells [20,21]. In order to survive, tumor cells have then to find new strategies to escape NK cells. Tumor-derived exosomes have been extensively associated with immunosuppression in cancer. In fact, multiple studies show that TDEs inhibit NK cytotoxicity (Figure 4A and Table 2) [119,120,121,122,123,124,125,126,127,128] and, thus, we believe that exosomes play a central role in tumor immune escape by preventing NK cell activation and tumor cell killing.

One of the most important activating receptors of NK cells is NKG2D [44,53]. Using a panel of tumor cell lines, Clayton et al. [119] found that TDEs express NKG2DLs. Furthermore, exosomes isolated from pleural fluid of three patients with malignant pleural mesothelioma also express NKG2DLs on their surface. Treatment of CD8^+^ T cells and NK cells derived from PBMCs of healthy donors with TDEs led to a decrease in NKG2D on the cell surface and, contrarily to what was expected, impaired their activation [119]. This phenomenon might be explained by the hypothesis that the lysosomal degradation of this receptor upon its internalization is being triggered by the persistent engagement of NKG2D receptor by its ligands, a mechanism reviewed in [40]. However, this hypothesis was not tested by the authors [119]. Moreover, the observed decrease in NKG2D mediated by TDEs was reversed by 41% on NK cells in the presence of neutralizing antibody against MICA [119], one of the NKG2DLs expressed on exosomes’ surface. Interestingly, neutralization of TGFβ1, an inhibitory cytokine, also inhibited NKG2D downregulation by 90% on human NK cells, suggesting that exosomal TGFβ1 and NKG2DLs cooperate to regulate NKG2D expression [119]. Similar results were observed for exosomes derived from human prostate cancer cells and from the serum of castration-resistant prostate cancer patients [121], as well as from human T cell leukemia Jurkat- and B cell leukemia/lymphoma Raji [120], HeLa cervical cancer [122] and L3.6pl pancreatic cancer [123] cell lines.

As evidenced by the fact that immunosuppressive TDEs can be found in the blood on cancer patients [121,125,126,127], the interplay between tumor cells and NK cells through exosomes might not be confined to the tumor site. An interesting study by Katsiougiannis et al. [124] tested whether salivary exosomes from Panc02 pancreatic tumor-bearing mice could modulate NK cells’ activity. The authors show that oral gavage of saliva from tumor-bearing mice into healthy mice, that were then intravenously injected with Panc02 cells to trigger an immune response, decreased the functional activity of spleen-derived NK cells and downregulated the expression of the activating receptors CD69 and NKG2D. In comparison, saliva from mice that were inoculated with Panc02 cells with a Rab11 downregulation and, thus, impaired exosomes production, did not inhibit NK cells’ function. Furthermore, incubation of mouse NK cells with salivary exosomes from tumor-bearing mice resulted in a marked reduction in NK cells’ ability to kill tumor cells [124].

In addition to NKG2D downregulation, other mechanisms of exosomes-mediated NK cells’ inhibition have been proposed. In fact, the expression of TGFβ1 on exosomes surface seems to be as important as the expression of NKG2DLs in the regulation of NK cells activity. This hypothesis is supported by the recent finding that clear cell renal cell carcinoma exosomes [145], as well as pancreatic cancer exosomes [123], interact with TGFβ receptors on human PBMCs-derived NK cells and human NK-92 cell line, respectively, activate the TGFβ-Smad2/3 pathway and, consequently, induce the phosphorylation Smad2/3, suppressing the activation of NK cells. Two studies by Hong et al. report that plasma exosomes from acute myeloid leukemia patients also express TGFβ1 and NKG2DLs, being able to inhibit human NK-92 cells’ proliferation, cytotoxicity and migration capacity and decrease the expression of NKG2D [126,127]. Recently, the same group reached similar conclusions upon incubation of melanoma patient-derived NK cells with exosomes isolated from the plasma of melanoma patients [125].

Another mechanism of tumor exosomes-mediated NK cells dysfunction has been proposed by Liu et al. [143]. Liu et al. observed that NK cells derived from the spleen of BALB/c mice pretreated with exosomes from murine mammary adenocarcinoma cell lines displayed reduced cytotoxicity against YAC-1 lymphoma cells [143]. In the same study, it was shown in vitro that mammary adenocarcinoma exosomes exert their inhibitory effect on NK cells through direct interaction. Furthermore, the authors observe a decrease in the expression of perforin in NK cells [143]. Perforin is released together with granzymes in the form of cytotoxic granules by NK cells, being responsible for the formation of pores in the membrane of target cells to allow the entrance of pro-apoptotic granzymes into their cytoplasm [128]. Given the importance of perforin for NK cells’ cytotoxic activity, the decreased expression of this protein is in line with the found impairment on NK cells’ ability to kill target cells.

Put together, these reports strongly suggest that tumor cells are able to release NKG2DLs-expressing exosomes that then are able to interact with the NKG2D receptors present on the surface of NK cells, as well as with CD8^+^ T cells, causing its internalization, hindering the NKG2D-dependent activation of these cells and providing immune escape to tumor cells. In addition, multiple studies observe that TGFβ1 plays a key role in TDEs’ ability to suppress NK cells-mediated tumor killing. Furthermore, TDEs also inhibit the expression of perforin in NK cells [143]. At last, evidence from different studies suggests that these immunosuppressive TDEs are able to travel to distant sites, being found in the blood of cancer patients [121,125,126,127] and saliva of tumor-bearing mice [124]. This raises the hypothesis that tumor cells might use exosomes to induce immunosuppression not only in the tumor site but also to suppress the proliferation and activation of immune cells in distant lymphoid organs, facilitating tumor progression and metastasis formation. In conclusion, this knowledge brings us a step closer to understand how tumor-derived exosomes mediate immunosuppression and how can we tackle this obstacle in the fight against cancer.

### 4.3. NK Cell-Derived Exosomes in Cancer Progression

Several studies suggest that exosomes released from NK cells might as well modulate the immune response, exerting effects in cancer progression. A study conducted in 2017 demonstrates that exosomes derived from primary NK cells, which were previously exposed to neuroblastoma cells, potentiate the antitumor activity of naïve NK cells, which acquire a memory-like phenotype (Figure 4B and Table 3) [146].

On the other hand, NK cell-derived exosomes can also be taken up by cancer cells and have a direct effect on cancer cells. In the context of neuroblastoma, human NK cell–derived exosomes were found to carry the tumor suppressor miR-186, which was shown to be responsible for the cytotoxic effects of NK-derived exosomes on neuroblastoma cells. In this study, Neviani et al. have shown that exosomal miR-186 directly downregulates MYCN and AURKA in neuroblastoma cells, which can potentially have a negative impact on their survival. Interestingly, miR-186 upregulation in NK cells resulted in the downregulation of TGFBR1 and TGFBR2 and, thus, impaired the TGFβ1-dependent inhibition of NK cells’ cytotoxicity. However, it is unclear whether the delivery of miR-186-expressing NK exosomes to NK cells could produce the same effects [147]. Another study conducted by Zhu et al. [148] showed that NK-92 cell-derived exosomes mediated anti-tumor effects against melanoma cells, decreasing their viability and proliferation. Furthermore, these exosomes presented FasL, perforin and TNF-α, which are well known to be involved in NK cell-mediated cancer killing. These findings were further corroborated by the in vivo experiments. In fact, tumors injected with NK cell-derived exosomes were smaller compared with those from the vehicle group [148]. Acute myeloid leukemia blast cells were also reported to be able to uptake exosomes derived from human NK cells, which, in turn, promoted in leukemia cells’ death. According to the authors, these NK cell-derived exosomes were loaded with NKG2D, TGFβ, granzyme B, perforin, killer-cell immunoglobulin-like receptors, and programmed cell death protein 1 (PD-1) [149]. Lugini et al. also observed that human PBMCs-derived NK cells-derived exosomes wielded similar cytotoxic activity against several human hematopoietic and solid malignancies in vitro [150]. Furthermore, Di Pace et al. showed that the cytotoxic activity of PBMCs-derived NK-derived exosomes against leukemia cells was dependent on DNAM1 present on the exosomes’ surface. This suggests that DNAM1 might assist the internalization of NK exosomes into tumor cells, with consequent release of its content to promote tumor cell lysis or induce apoptotic pathways. LFA-1 was also detected at the surface of NK cell-derived exosomes; however, it did not seem to play a role in NK cell’s exosomes-mediated cytotoxicity [151]. Last year, Chun-Hua Wu and colleagues demonstrated that human NK cell-derived exosomes contained cytotoxic proteins and activated caspases, inducing target cells’ apoptosis. The exosomal content included perforin, FasL, granzyme A and B, and granulysin. These findings indicated that there are multiple killing mechanisms activated by NK cell-derived exosomes, thus mediating cytotoxicity against tumor cells [152] and corroborating the previous findings. A recent study by Federici et al. [153] confirmed that exosomes derived from NK cells are enriched in proteins implicated in the immune response. They also showed that exosomes derived from NK cells are involved in T and NK cells’ activation. Nevertheless, these effects were more pronounced when T and NK cells were treated with microvesicles derived from NK cells instead. Furthermore, this study also shows that, in the presence of either exosomes or microvesicles from NK cells, there is a decrease in PD-1 expression in T cells [153].

Altogether, these findings suggest the potential of using NK cell-derived exosomes as an immunotherapeutic strategy for several types of cancers, including neuroblastoma, melanoma, and leukemia. Nevertheless, the potential of other EVs, such as microvesicles, in the regulation of the immune system should also be considered and explored further.

**Table 1:** Tumor exosomes as mediators of NK cells activation.

Exosomes Origin	Exosomes’ Characteristics	Effect on NK Cells	Reference
Human pancreatic (Colo357) and colon (CX2) cancer cell lines	Hsp70/Bag4-positive exosomes	Stimulation of the migratory capacity and cytolytic activity of NK cells	[112]
Melanoma cell line Ge	Hsp70-expressing exosomes	Stimulation of the migratory capacity and cytolytic activity of NK cells; NK cells activated by TDEs were prone to eliminate YAC-1 cells, which expressed NKG2DLs in a constitutively manner, and NKG2DL-overexpressing human melanoma cells.	[113]
Human hepatocarcinoma HepG2 cells	Expression of Hsp70, Hsp90 and Hsp60 on exosomes’ surface	Stimulation of NK cells’ cytotoxicity (measured by the enhanced expression of CD69, NKG2D, and NKp44 receptors and downregulation of CD94 on the surface of NK cells, as well as by the release of granzyme B)	[114]
Engineered myeloma J558 cell line	Hsp70-expressing exosomes	Boost CD8+ T cells’ and NK cells’ cytotoxicity in BALB/c mice inoculated with this cell line and protected all mice injected subcutaneously with J558 mouse myeloma cells from developing tumor.	[115]
SKO-007-subclone J3 myeloma cells	Hsp70-expressing exosomes	Activation of the TLR2/NFκB signaling pathway through Hsp70 and induction of cytokine IFN-γ production	[140]
Multiple myeloma cells	IL-15/IL-15Rα-expressing exosomes	Unknown	[144]
K562 myeloid leukemia cells	Exosomes engineered to overexpress IL-15, IL-18, and 4-1BBL	NK cells treated with these exosomes showed enhanced killing activity, release of perforin and production of IFN-γ.	[118]

**Table 2:** Tumor exosomes as mediators of NK cells suppression.

Exosomes Origin	Exosomes’ Characteristics	Effect on NK Cells	Reference
Pleural fluid from patients with malignant pleural mesothelioma	Expression of NKG2DLs on exosome’s surface	Decrease in NKG2D on NK cells’ surface and impaired NK cells’ activation	[119]
Human prostate cancer cells; serum of castration-resistant prostate cancer patients; human T cell leukemia Jurkat- and B cell leukemia/lymphoma Raji cell lines; HeLa cervical cancer; L3.6pl pancreatic cancer cell lines	Expression of NKG2DLs on exosome’s surface	Decrease in NKG2D on NK cells’ surface and impaired NK cells’ activation	[120]
Saliva of Panc02 pancreatic tumor-bearing mice	N/A	Decreased the functional activity of spleen-derived NK cells and downregulated the expression of the activating receptors CD69 and NKG2D; reduced NK cells’ ability to kill tumor cells.	[124]
Clear cell renal cell carcinoma; pancreatic cancer	Expression of TGFβ1 on exosomes surface	TDEs interact with TGFβ receptors on NK cells, activate the TGFβ-Smad2/3 pathway in NK cells and, consequently, induce the phosphorylation of Smad2/3, suppressing the activation of NK cells	[123,145]
Plasma of acute myeloid leukemia patients	Expression of TGFβ1 and NKG2DLs on exosomes surface	Inhibition of NKs’ proliferation, cytotoxicity and migration and decrease the expression of NKG2D	[126,127]
Murine mammary adenocarcinoma cell lines	N/A	Reduced the cytotoxicity of spleen-derived NK cells against YAC-1 lymphoma cells; decrease in the expression of perforin in NK cells.	[143]

**Table 3:** NK cell-derived exosomes in cancer progression.

NK Cell-Derived Exosomes’ Characteristics	Effect on Cancer Progression	Reference
Carry tumor suppressor miR-186	Directly downregulates MYCN and AURKA in neuroblastoma cells, which can potentially have a negative impact on their survival; miR-186 upregulation in NK cells resulted in the downregulation of TGFBR1 and TGFBR2 and, thus, impaired the TGFβ1-dependent inhibition of NK cells’ cytotoxicity.	[147]
Presented FasL, perforin and TNF-α	Decreased melanoma cells’ viability and capacity of proliferation	[148]
Exosomes loaded with NKG2D, TGFβ, granzyme B, perforin, killer-cell immunoglobulin-like receptors and PD-1	Promote the lysis of acute myeloid leukemia blast cells	[149]
N/A	NK cell-derived exosomes presented T cell leukemia, Burkitt lymphoma, metastatic breast adenocarcinoma, and metastatic melanoma human tumor cell lines	[150]
Expression of DNAM1	Cytotoxic activity of NK-derived exosomes against leukemia cells	[151]
Expression of perforin, FasL, granzyme A and B, and granulysin	Induction of target cells’ apoptosis	[152]

## 5. Conclusions

Intercellular communication is essential for cancer progression, metastasis establishment and drug resistance acquisition, potentiating changes in the tumor landscape, which allow tumor immune escape. Exosomes are deeply involved in the intercellular communication between tumor cells and the TME, and have a preponderant action in the modulation of the TME cells for tumor benefit. Here, we highlight that TDEs have an active role in NK cells regulation, either by stimulating or inhibiting their cytotoxic activity. NK cells-derived exosomes were also shown to be important players in the regulation of NK cells’ function, as well as in cancer killing. Nonetheless, most studies that show that TDEs and NK cell-derived exosomes regulate NK cells’ activity were performed in vitro or ex vivo. Thus, it is still unclear where these interactions take place. Furthermore, although it would be expected that most of the interactions between TDEs and NK cells would take place locally (within the TME or in local lymphoid organs), evidence also shows that TDEs can be found in circulation and in mice saliva [121,124,125,126,127], suggesting that they might have a role on NK cell development and recruitment, as well as on the invasion of distant organs by tumor cells. Thus, it is of utmost importance that an effort is made in future studies to address these points using in vivo models, and validating the findings in human material. Altogether, the effects of TDEs and NK cell-derived exosomes that have been so far reported in the literature open an avenue for using exosomes as an immunotherapeutic approach, either by targeting them, or using them as nanocarriers. Additionally, engineered exosomes could be used to modulate the immune system, namely NK cells. This could improve the clinical response to immunotherapy and other therapeutic approaches for cancer treatment, augmenting the life expectancy of cancer patients.

## Abbreviations

ADCC	Antibody-dependent cell-mediated cytotoxicity
APC	Antigen Presenting Cell
BAG	BCL-2-associated-athanogene
CLP	Common Lymphoid Progenitor cells
DC	Dendritic Cell
DNAM-1	DNAX Accessory Molecule-1
ESCRT	Endosomal Sorting Complex Required for Transport
EV	Extracellular Vesicle
FasL	Fas Ligand
HSC	Hematopoietic Stem Cell
HSP	Heat shock protein
IFN	Interferon
IL	Interleukin
ILV	Intraluminal Vesicle
LFA-1	Lymphocyte Function-Associated Antigen-1
lncRNA	long non-coding RNA
MCMV	Murine Cytomegalovirus
MDSC	Myeloid-Derived Suppressor Cell
MHC I	Major Histocompatibility Complex I
MHC II	Major Histocompatibility Complex II
miRNA	micro-RNA
mNK	Mature Nature Killer cell
MVB	Multivesicular Body
NCR	Natural Cytotoxicity Receptor
NFκβ	Nuclear factor-kappa β
NK	Natural Killer
NKG2D	Natural Killer Group 2 member D
NKG2DL	Natural Killer Group 2 member D Ligand
PBMCs	Peripheral Blood Mononuclear Cells
PD-1	Programmed Cell Death Protein-1
PDGF-DD	Platelet-Derived Growth Factor-DD
TDE	Tumor Derived Exosome
TGFβ	Transforming Growth Factor β
TLR	Toll-like receptor
TME	Tumor Microenvironment
TNF	Tumor Necrosis Factor
Treg	Regulatory T cell
VSV	Vesicular Stomatitis Virus
WHO	World Health Organization
